# Is Aesthetic Good? A Study on the Aesthetic and Vitality Judgment of Pictorial Representations of the Dead, Saints and Non-Saints

**DOI:** 10.3390/bs12120507

**Published:** 2022-12-13

**Authors:** Sara Valentina Schieppati, Cinzia Di Dio, Antonella Marchetti, Davide Massaro, Gabriella Maria Gilli

**Affiliations:** 1 Research Unit of Psychology of Art, Department of Psychology, Università Cattolica del Sacro Cuore, 20123 Milan, Italy; 2 Research Unit of Theory of Mind, Department of Psychology, Università Cattolica del Sacro Cuore, 20123 Milan, Italy

**Keywords:** aesthetic, vitality judgment, paintings, Saints, religious art, psychology of art

## Abstract

In the history of the Western world, there has always been an association between good and beautiful. Starting from a brief history of beauty, two questions arise: is beauty linked to good even in art? How important are people’s religious beliefs in aesthetic and vitality judgments? The psychology of art could answer these questions by studying people’s reactions to the images of Saints as testimonials of goodness. Moreover, the study of Saints’ paintings would allow us to investigate vitality, understood as one’s perception of a living being. The research aimed to investigate the aesthetic and vitality judgments of faces representing the dead, Saints and non-Saints. More than a hundred participants were asked to evaluate the aesthetics and vitality of these paintings; moreover, two tests assessing spirituality and religiosity were administered. Overall, these data suggest Saints were judged more beautiful than non-Saints, and non-Saints were judged more vital than Saints. This might suggest a relationship between ethics and aesthetics, also in the perception of art, and offers reflections on the theme of vitality. The religion and spirituality of participants are not correlated to aesthetic or vitality judgments; this fact could support that these judgments are linked to the basic bottom-up reactions to images.

## 1. Introduction

The stereotype “what is beautiful is good” has been advocated in different societies and eras in the Western world. Already in the Iliad, the gods and heroes are characterized by having a positive epithet. For example, Achilles is depicted as “with beautiful hair”; conversely, the anti-heroes are characterized by physical distortions, for example, Thersites (the anti-hero par excellence) is described, among other things, as lame in one foot [1]. These are probably primordial traces of kalokagathìa, an expression that would be born in the 5th century BC in Athens, which indicates an ideal of unity between visible physical beauty (kalòs) and (kai), and moral quality (agathòs) [2,3]. The concept of kalokagathia has spread in different fields, from philosophy, with the aesthetics of Plato, to art, with the use of the golden section in painting and sculpture. The Romans received the cultural heritage of the Greeks and this idea of harmony between mind and body continues to influence artistic production [2]. For example, the Roman statues, while differing from the Greek statues because of their greater realism, are still built using the golden section. Starting from late antiquity, the Greco-Roman culture was enslaved to Christianism: physical beauty, although not despised, is temporary and therefore is considered inferior to moral beauty; however, moral beauty is reflected through the body, and therefore beauty is still associated with virtue, and ugliness with vice [4,5]. For this reason, in the Middle Ages and the Renaissance, it is possible to find depictions of martyrs and Saints (for example Sant Irene) who are represented as virtuous and beautiful [2,6]. From the Enlightenment onwards, the theme of beauty is disconnected from the theme of religion and is regarded differently according to the field of reference; there is no longer a universal beauty, but there is a subjective beauty that depends on the observer and on the field from which it is investigated [2,4]. Thus, since the Enlightenment, we have passed through epochs exalting the idea of beauty linked to art, then to nature, and then to subjectivity; with the industrial revolution and the advent of the reproducibility of artworks, the theme of beauty is set aside [2,7]. In the Contemporary era, it seems that the two currents, that of the Greek–Roman tradition and that of the Enlightenment tradition, collide, and therefore these two questions arise: is beauty linked to good even in art? Equally, how important are people’s beliefs in aesthetic attribution?

Psychology, and especially social psychology, has investigated the relationship between beauty and good through numerous studies showing that people derive, on the one hand, moral inferences from physical beauty and, on the other hand, information relating to morality’s influence upon aesthetic judgments [8,9,10]. However, of all this research, the psychology of art has so far provided no significant additions, although it could contribute to the debate with interesting studies. Research into Saints’ paintings could offer fascinating insights. From the Middle Ages onwards, the paintings of the Saints were proposed to offer devotees models of beautiful and virtuous men [2,4], as can be seen in the representations of St. Sebastian (e.g., San Sebastiano of Botticelli) [11]. However, are Saints perceived as beautiful? There is still no research that has evaluated the aesthetics of the Saints, and yet, knowing how Saints are perceived from an aesthetic point of view would allow us to answer both the question of whether moral beauty is linked to physical beauty, and whether people’s beliefs influence aesthetic evaluation. In brief, if Saints were perceived as aesthetically beautiful, especially by religious people, this fact, on the one hand, would support the relationship between beauty and good, and on the other hand, would confirm that religious beliefs influence aesthetic perception in religious art. The study of Saints’ paintings would also allow us to investigate another little-explored theme in the field of the psychology of art: vitality. There is no precise definition of what vitality is in images, so it is very complex to deal with this theme; however, this concept could be understood as a force that resides within the image that allows the viewer to see the images as if they were alive, and that would depend on both the image itself and on the viewer. It is not a question of factuality, but it is about the reactions to the image and its perception. In the Western world, and particularly in the Catholic world, which encouraged visual art for religious purposes [12], there are various accounts of “vital” artworks, such as statues or paintings that cry or move their eyes [13,14]. In the “Dialogus magnus visionum et miraculorum” by Cesario di Heisterbach, it is possible to find hagiographic stories that also involve art and images. For example, there is a story of a portrait of St. Nicholas, a protector of pregnant women, who, placed in front of a woman in childbirth, turns towards the wall to avoid looking at the birth [13,15]. Interestingly, according to Freedberg [13,16], it is precisely the features of the face (especially the eyes) that give vitality to an image. According to psychology, the face provides fundamental information for interactions between humans [17], and also between humans and robots [18]. However, if it is true that the face is fundamental in everyday interactions, it also seems to be so in images, according to studies on iconoclasm [19,20,21,22]. Iconoclastic movements have often attacked the face, as if the image to be scarred were a real person, as happened, for example, to the painting “Seven Works of Mercy”, by Master of Alkmaar, where the eyes were intentionally targeted. This artwork was damaged during the iconoclastic movements of 1566 when Protestants vandalized Catholic churches [21,22]. Therefore, the vitality of the images is expressed through the face, but from here at least three questions arise. Firstly, is there a difference between religious and secular images? The living images reported by the tales often refer to paintings depicting Saints [13], and therefore it would be interesting to verify if religious images have greater vitality than other types of images. Secondly, does vitality fail if the subjects are represented as dead and with their eyes closed? Practically, it would be interesting to understand whether the attribution of vitality to images could persist even in the absence of the strongest elements of vitality, the eyes [13]. Finally, what relationship, if any, exists between aesthetics, vitality, and beliefs? With these questions in mind, we aimed to investigate whether an image perceived as alive is also seen as beautiful (and vice versa, an image perceived as not very lively is seen as unattractive) [17], and if this relationship can be influenced by religiosity and/or spirituality. The present research aims to investigate the aesthetic evaluation and judgement of vitality in images of faces representing dead Saints and non-Saints. Based on the theoretical background introduced above, it was hypothesized that participants would attribute a higher aesthetic evaluation and a higher vitality judgment to images of dead Saints than dead non-Saints. It was also hypothesized that religious status and spirituality would have some influence on the aesthetic evaluation and the vitality judgment of the image; namely, Catholics and highly spiritual people would express a greater aesthetics and vitality judgment compared to non-Catholics and poor spiritual people. Our hypotheses have been only partially confirmed: the Saints are more beautiful but no more vital than the non-Saints, and there is no correlation between aesthetic and vitality judgments, and the religious and spiritual dimensions.

## 2. Materials and Methods

### 2.1. Participants

The study involved 114 Italian participants, aged between 18 and 52 years (M = 24.2; SD = 5.40), almost half females (54.4%). About 58% of participants had a high school diploma, and many (27.2%) had a bachelor’s degree. Most of the participants (92.9%) did not possess specific artistic skills or knowledge; moreover, 60.5% declared that they visited museums or galleries twice a year and 21% did not frequent these places at all. Additionally, most of the participants reported being non-religious (42.1%), whereas 30.7% were religious, and 27.2% were uncertain about their faith; in addition, they were asked to indicate the religious context that they recognize as a cultural reference. By cross-referencing this information, a dichotomous variable was created, religious status. On the one hand, Catholics, that is, religious and uncertain people who use Catholicism as a reference, and, on the other hand, the Others, that is, non-religious, uncertain, and religious people who refer to a religion other than the Catholicism. Concerning religious status, participants were divided into two categories: Catholics (52.6%) and Others (47.4%), that is, people who did not recognize themselves in any religion or recognize a religion other than Catholicism.

### 2.2. Design and Procedure

Through the psychology department, using snowball sampling, an email was sent with the invitation and link to access the research. Participation in the study, which took approximately 15–20 min, was voluntary and participants gave their written consent. Demographic data, artistic competence, and religious status were collected first. Then, the participants were asked to assign an aesthetic and vitality evaluation to the images presented in a randomized order. They were subsequently asked to indicate whether the faces were of Saints or non-Saints, and to indicate how familiar they were with the images before the study. Finally, they were administered two tests: the Spirituality Assessment Scale [23] in the Italian version [24], and the Utrecht-Management of Identity Commitments Scale (U-MICS; [25]) to assess spirituality and religiosity, respectively.

### 2.3. Measures

#### 2.3.1. Aesthetics and Vitality

The selection of the stimuli was carried out through online art galleries (Web Gallery of Art, Freeart—online art museum, Hermitage Museum—online collection). The images were included in the study if they met the following criteria: (a) the subjects are represented as dead; (b) the subjects show no signs that could immediately identify them as dead (e.g., wounds or dull skin); (c) the subjects have their eyes closed; (d) the artworks considered are dated between the early 1300s and early 1700s. From the initial selection that met these criteria (about 130 images), after discarding those images that were impossible to manipulate without noticeable distortions (e.g., removal of a large portion of the face to hide the nimbus) or impossible to isolate (e.g., individual portrayed in a crowd), the 8 images with the best sharpness and resolution were selected (4 of Saints and 4 of non-Saints). The final number of images presented was based on previous research [17], showing that a comparison of eight images divided into two categories was sufficient to detect any significant variations in beauty and vitality. The faces were cut out of the original painting using GIMP so that all contextual elements were removed, and the stimuli were made homogeneous in terms of resolution (300 × 300 px) (Table 1).

For each image, a pair of adjectives (ugly–beautiful; dead–alive) was presented and the participants were asked to indicate in each pair the position that best described their opinion on a 7-point scale to measure the aesthetic evaluation (1 = ugly; 7 = beautiful) and the judgment of vitality (1 = dead; 7 = alive).

#### 2.3.2. Familiarity

Several studies show that being familiar with a stimulus makes it more attractive, and this also happens for paintings and faces; if already known, they are perceived as more beautiful [26,27]. For this reason, the participants were re-proposed the same images and asked to indicate, on a 5-point scale (1 = not at all; 5 = a lot), how familiar they were with the artworks before the study.

#### 2.3.3. Recognition

Participants were asked to indicate whether the image presented was the face of a Saint or non-Saint. This information potentially makes it possible to link correct or incorrect recognition with religious status.

#### 2.3.4. Spirituality

Spirituality can be defined as “the human desire for transcendence, introspection, interconnectedness, and the quest for meaning in life” [28] (p. 3). Spirituality was measured through the Italian version [24] of the Spirituality Assessment Scale [23]. This scale has 28 items, rated on a 6-point scale (1 = strongly disagree; 6 = strongly agree), and is divided into four subscales that measure the different dimensions of spirituality: Scope (4 items), Interiority (9 items), Interconnection (9 items), and Transcendence (6 items).

#### 2.3.5. Religiosity

Religiosity was investigated through the Italian-validated version of the Utrecht-Management of Identity Commitments Scale (U-MICS) [25] which measures three dimensions of religious identity: Commitment (5 items), In-depth exploration (5 items), and Reconsideration of the commitment (3 items). People make commitments related to their religious identity by adhering, for example, to a specific vision of reality, and then they can decide to strengthen their commitment or reduce it and explore other beliefs [24,25,26,27,28]. Items were measured on a 5-point scale (1 = completely false; 5 = completely true). This scale was only administered to participants who had defined themselves as religious or uncertain, since non-religious people could not answer questions related to their religion.

## 3. Results

### 3.1. Exploratory Correlation Analyses

Pearson’s correlation analyses were carried out to evaluate the relationship between the aesthetic and vitality judgment of Saints and non-Saints, and the participants’ age, gender, level of education, attendance of museums or galleries, religious status, and self-reports of familiarity with the stimuli. The analysis showed a significant relationship (Bonferroni corrected for multiple comparisons, *p* < 0.01) between familiarity and aesthetic judgment, r = 0.25, and familiarity and vitality judgment, r = 0.27, for the dead Saints only. This effect was plausibly influenced by religious status which correlated with greater familiarity with the images of Saints, r = 0.23

### 3.2. Recognition

In line with the results above, when asked to recognize the images by indicating if they portrayed a Saint or non-Saint, recognition indicated that participants recognized most images—except for two (one Saint and one non-Saint) above chance level, *p* < 0.05. The percentage of correct recognition is reported in Table 2. Contrary to the results above, this was independent of the participant’s religious status.

### 3.3. Aesthetic and Vitality Judgment

**Figure 1:**
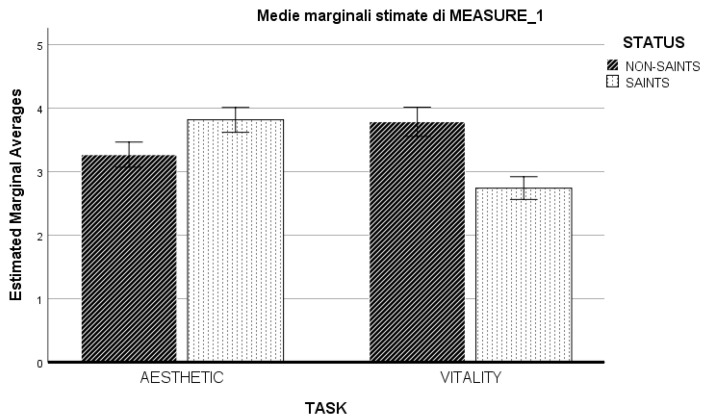
Bar graph showing the interaction between task (Aesthetic and Vitality judgment) and stimulus category (Saint, striped bars; non–Saint, dotted bars). Error bars represent 95% confidence intervals.

To evaluate the differences in the aesthetic and vitality judgments between images of Saints and non-Saints, a GLM repeated measures analysis was carried out with two levels of task (aesthetic, vitality), and two levels of image category (saints, non-saints) as within-subjects factors. As familiarity positively correlated with the aesthetic and vitality judgments for Saints’ portraits, familiarity was included in the model as a covariate. Post hoc comparisons were Bonferroni corrected. The results revealed a significant interaction between task and category (Figure 1), F(1, 112) =16.31, *p* < 0.001, partial-η2 = 0.13, δ = 0.98, indicating that Saints were judged more beautiful than non-Saints, Mdiff = 0.55; SE = 0.12, *p* < 0.001, and that non-Saints were judged more vital than Saints, Mdiff = 1.04; SE = 0.13, *p* < 0.01. No significant interactions were found between any of the factors and the covariate familiarity, *p* > 0.05.

### 3.4. Spirituality and Religiosity

We carried out Pearson’s correlation analyses (Bonferroni corrected, *p* < 0.01) to evaluate if the religious and/or spiritual dimensions were associated with aesthetic and vitality judgments of the Saint and non-Saint images. As religious people may be quite familiar with religious art, thus affecting the aesthetic judgment of images of Saints, correlations were further carried out between familiarity with the stimulus and religiosity. No associations were found between the religious and/or the spiritual dimensions and the aesthetic and vitality judgments, *p* > 0.05. Similarly, no significant associations were found between religiosity and familiarity for both the Saints and non-Saints, *p* > 0.05.

## 4. Discussion

This study aimed to evaluate differences in the aesthetic and vitality judgments for images showing pictorial representations of Saint and non-Saint dead people. The effect of religiosity and spirituality on such judgments was also explored. The results showed that Saints were judged more beautiful than non-Saints, and that non-Saints were judged more vital than Saints, thus only partially supporting our hypothesis. These differences appear not to be related to either the participants’ religious and/or spiritual inclinations or the contextual dimension (i.e., catholic vs. other religious or atheists). These results open several reflections.

Firstly, the Saints were perceived as more beautiful than non-Saints. This result is not related to the individual’s religiosity or spirituality, given the absence of a correlation between the religious/spiritual sphere and the aesthetic judgment of Saints. Furthermore, this result cannot be justified in terms of greater familiarity with the images, because in the analysis of the differences between the images of Saints and non-Saints, we took into consideration familiarity and no significant correlations emerged; moreover, no correlation emerged even between familiarity and religious dimension or context. What, then, could be a possible explanation for the aesthetic preference ascribed to Saints? Focusing on the intrinsic value of the religious image, a possible interpretation can be traced back to the connection between the beautiful and good, as described in the introduction: Saints are perceived as attractive due to a vague halo effect [29,30]. The halo effect is a cognitive bias in which the perception of a trait is influenced by the perception of one or more different characteristics of the person [31]. For example, physical attractiveness is linked to positive inferences about personality, and therefore a beautiful person can be perceived as good and a good person can be perceived as beautiful [32]. This is what happens in our case: Saints are recognized as such, that is, as virtuous people, and hence they are also considered beautiful.

Additionally, opposite to our initial predictions, the non-Saints were regarded as more vital than the Saints. A possible explanation could be based on intrinsic pictorial differences between the two groups of stimuli (Saints and non-Saints). However, we would tend to exclude this hypothesis because, if we consider the styles, the historical period of creation, and the painting definition that made clear the signs of death vitality (e.g., closed eyes, yellowish skin, etc.), the two groups of stimuli tend to balance. However, it cannot be ruled out that vitality depends on the intentionality of the painters. In an interpretative key, therefore, turning the perspective, we can say that Saints are less vital than non-Saints. We could argue that the Saints are perceived as more spiritual and distant from everyday life, and therefore less vital if one conceives vitality as a corporeal and concrete quality. The problem here lies precisely in the lack of a shared definition of vitality, and a solution must be found to continue the studies of this concept from a psychological point of view. To refine a definition, it might be interesting to do qualitative and quantitative research and, for example, ask people what makes a painting vital to them and if there is any correspondence between vitality and the reactions and perceptions of the paintings. Moreover, it should be investigated whether this form of vitality is different from the vitality investigated by neuroscience [33].

Another significant result is that the religious status and the spirituality of the participants do not correlate with either the judgment of aesthetics and vitality, or the correct recognition of the images. This fact could support the idea that there is always a basic level of reaction to images that is independent of the cultural context [12,13], and therefore the judgments of vitality and aesthetics are linked to the basic bottom-up reactions to images and are not significantly influenced by top-down socio-cultural elements, such as religiosity and spirituality.

## 5. Conclusions

The results of this study show a relationship between the beautiful and good in the perception of religious art images. As reported in the scientific literature, e.g. [26,32], not only do aesthetics influence moral perception, that is, whoever is beautiful is also seen as good, but the opposite is also true, that whoever is perceived as an ethically correct person is also seen as more beautiful. Moreover, it is interesting to note that spirituality and religiosity, which we assumed before the study to be two relevant variables in aesthetic and vitality judgments, actually have no bearing in this regard. Therefore, it would be interesting to carry out this research in countries where the Catholic religion is more strongly felt, to investigate whether these variables are irrelevant. In addition, it would be interesting to have two groups, one Catholic and one Protestant, for example, to see if there is any change in the perception of religious images, given that these two religious currents have very strong differences in the acceptance and the use of religious images [12]; in this case, it would be possible to better understand the respective weight of the top-down and bottom-up variables. A possible future extension of this area of research could involve the application of this research design to the study of imagery in Byzantine art, which could be particularly interesting because it involves not only the topics of vitality and holiness, but also the theme of iconoclasm [13,20]. Moreover, it could be very useful to have a comparison between art experts and non-art experts to highlight possible socio-cultural influences on the perception of these images. Lastly, for reruns or future extensions of this research, the inclination of the head in the paintings should be controlled to understand if this variable could have any influence on the recognition of the Saints. In fact, in religious figures, including the Virgin and Saints, the canting of the head is a sign of submission and devotion. For example, nobles in images have slightly less inclination than the Saints [34]. 

In conclusion, this work offers an important initial starting point for investigating, outside of the religious domain, the intrinsic nature between the good and the beautiful, and therefore between ethics and aesthetics; this is a theme that has already been extensively dealt with over the centuries in the philosophical sphere, but that could also be further explored in the psychological field. Finally, this study is vital and pioneering, in that it opens up several research questions that will require adequate and in-depth studies.

## Figures and Tables

**Table 1:** Description of the paintings from which the selected stimuli were extracted: author, title, year, museum, cropped stimuli.

Author	Title	Year	Museum	Cropped Stimuli
Cristofano Allori	Judith with the head of Holofernes	1571–1621	Pitti Palace, Florence	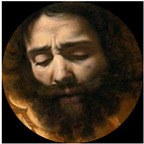
Peter Paul Rubens	The death of the consul Publius Decius	1616–1617	Museo del Prado, Madrid	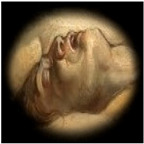
Lucas the Younger Cranach	Portrait of Martin Luther on his Deathbed	1546	Niedersächsisches Landersmuseum, Hannover	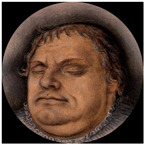
Cornelis Holsteyn	Venus and Amor mourning the death of Adonis	c.1655	Frans Hals Museum, Haarlem	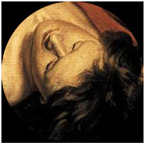
Ludovico Carracci	Saint Sebastian thrown into the Cloaca Maxima	1612	Getty Museum, Los Angeles	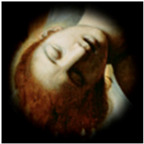
Daniele da Volterra	The beheading of St. John the Baptist	c.1555	Galleria Sabauda, Turin	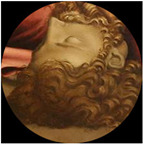
Georges de La Tour	Discovery of body of St Alexis	c.1649	Musée Historique Lorrain, Nancy	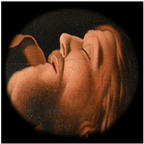
Serafino Serafini	Death of St Louis	c.1393	Church of San Francesco, Mantua	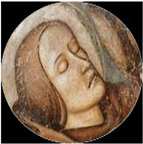

**Table 2:** Summary of contingency tables indicating the correct recognition (%) for religious status. * Indicates correct recognition above chance level, *p* < 0.05.

Cropped Stimuli	Religious Status	
	Catholic	Other
Judith with the head of Holofernes	38.3% *	38.9%
The Death of the consul Publius Decius	63.3% *	81.5% *
Portrait of Martin Luther on his Deathbed	93.3% *	94.4% *
Venus and Amor mourning the death of Adonis	81.7% *	83.3% *
Saint Sebastian thrown into the Cloaca Maxima	40%	37%
The beheading of St. John the Baptist	71.7% *	74% *
Discovery of body of St Alexis	75% *	83.3% *
Death of St Louis	91.7% *	77.8% *

## Data Availability

All data needed to evaluate the conclusions in the article are present in the article.
